# Role of p97/VCP (Cdc48) in genome stability

**DOI:** 10.3389/fgene.2013.00060

**Published:** 2013-04-30

**Authors:** Bruno Vaz, Swagata Halder, Kristijan Ramadan

**Affiliations:** ^1^Institute of Pharmacology and Toxicology, University Zürich-VetsuisseZürich, Switzerland; ^2^Gray Institute for Radiation Oncology and Biology, Department of Oncology, University of OxfordOxford, UK

**Keywords:** p97/VCP, Cdc48, genome stability, DNA damage response, DNA replication, DNA repair, chromatin-associated protein degradation, protein-induced chromatin stress

## Abstract

Ubiquitin-dependent molecular chaperone p97, also known as valosin-containing protein (VCP) or Cdc48, is an AAA ATPase involved in protein turnover and degradation. p97 converts its own ATPase hydrolysis into remodeling activity on a myriad of ubiquitinated substrates from different cellular locations and pathways. In this way, p97 mediates extraction of targeted protein from cellular compartments or protein complexes. p97-dependent protein extraction from various cellular environments maintains cellular protein homeostasis. In recent years, p97-dependent protein extraction from chromatin has emerged as an essential evolutionarily conserved process for maintaining genome stability. Inactivation of p97 segregase activity leads to accumulation of ubiquitinated substrates on chromatin, consequently leading to protein-induced chromatin stress (PICHROS). PICHROS directly and negatively affects multiple DNA metabolic processes, including replication, damage responses, mitosis, and transcription, leading to genotoxic stress and genome instability. By summarizing and critically evaluating recent data on p97 function in various chromatin-associated protein degradation processes, we propose establishing p97 as a genome caretaker.

## INTRODUCTION

Genome stability is a prerequisite for cell survival, cancer prevention, and control of aging (Papamichos-Chronakis and Peterson, 2013). The genome is constantly attacked by various reactive oxygen species from endogenous sources. In addition to endogenous sources, DNA lesions can also be generated by a variety of exogenous sources, such as ionizing radiations (IR), the ultraviolet light (UV), and many chemical agents, some of which are products of our industrialized society. It is estimated that the mammalian genome accumulates thousands of DNA lesions every day that disturb DNA synthesis and cell division, two essential processes in genome amplification, preservation and transition to the next generation (Jackson and Bartek, 2009). Failure to maintain genome stability leads to chromosomal aberrations, gene mutations, and cancer or cell death. To maintain genomic stability, cells respond to DNA damage by activating a spatiotemporal signaling pathway known as the DNA damage response (DDR; Ciccia and Elledge, 2010). Following DNA damage, the DDR drives cell cycle checkpoints and initiates DNA repair or induces apoptosis if the genome is severely damaged. Although DDR after double-strand break (DSB) has been widely investigated and is considered as a DDR model, other types of DNA lesions can cause activation of lesion-specific DDRs that signal and recruit appropriate sensor, transductor, and effector proteins (Jackson and Bartek, 2009). For example, unrepaired DNA lesions that enter S phase encounter a serious problem during DNA replication. When the DNA replication fork approaches these lesions, the cell activates the DNA damage tolerance (DDT) pathway, which enables survival by activating the translesion DNA synthesis (TLS) pathway (Friedberg, 2005). The TLS pathway enables recruitment of translesion DNA polymerases (DNA pols), which can bypass bulky DNA lesions and ensure continuous DNA synthesis. Activation of lesion-specific DDR and appropriate DNA repair mechanisms or DDT ensures cell survival and prevents genomic instability and cancer. DDR and DDT are controlled by various post-translational modifications (PTMs) in which ubiquitination and sumoylation play an essential role (Bergink and Jentsch, 2009; Al-Hakim et al., 2010; Ulrich and Walden, 2010; Bekker-Jensen and Mailand, 2011; Lehmann, 2011b; Psakhye and Jentsch, 2012). Ubiquitination and sumoylation regulate timely assembly and disassembly of various DNA repair and genome caretaker molecules. Disturbances in this tight regulation could cause severe defects in the DDR and lead to genomic instability, which has been demonstrated in certain types of breast and ovarian cancers and patients with RIDDLE syndrome (Blundred and Stewart, 2011; Lipkowitz and Weissman, 2011). Mutations in BRCA1, an E3 ubiquitin ligase involved in DDR, can result in breast and ovarian cancers, while mutations in the DDR-related E3 ligase RNF168 can cause RIDDLE syndrome, which is characterized by radiation hypersensitivity, immunodeficiency, dysmorphic features, and learning difficulties. Because many chromatin-associated DDR proteins are tightly bound to sites of DNA damage and are often protected from degradation, spatiotemporal turnover and degradation are facilitated by the ubiquitin-proteasome system (UPS; Dianov, 2011; Levy-Barda et al., 2011; Ramadan and Meerang, 2011). It is still not known how the UPS and its largest component, the proteasome, approach, remove and degrade tightly bound protein complexes on chromatin. Discovery of a p97 function related to chromatin may provide the answer (Dantuma and Hoppe, 2012; Ramadan, 2012).

In recent years, ubiquitin-dependent molecular chaperone p97 has emerged as an essential regulator of UPS-dependent protein turnover in chromatin. p97, also known as valosin-containing protein (VCP) or Cdc48, a central element of the UPS and is integrated downstream of substrate ubiquitination and upstream of the proteasome. With intrinsic ATPase activity, p97 extracts (segregates) polyubiquitinated proteins from diverse cellular locations and presents them for proteasomal degradation. p97 is also involved in degradation of highly folded ubiquitinated soluble proteasome substrates and thus functions as unfoldase (Beskow et al., 2009). Recently elucidated functions of p97 in several DNA metabolic processes indicate that the protein is a constitutive component of various essential chromatin-related processes in the cell cycle, DNA replication and repair, mitosis, and transcription. However, the function of p97 as it relates to genome stability has not yet been esteemed.

In this review, we discuss various p97 functions in chromatin-related processes essential for maintenance of genome stability and attempt to establish p97 as a genome caretaker. We also present a large body of evidence suggesting that protein-induced chromatin stress (PICHROS) induces genotoxic stress, ultimately leading to genome instability.

## UBIQUITIN-DEPENDENT MOLECULAR CHAPERONE p97

**FIGURE 1:**
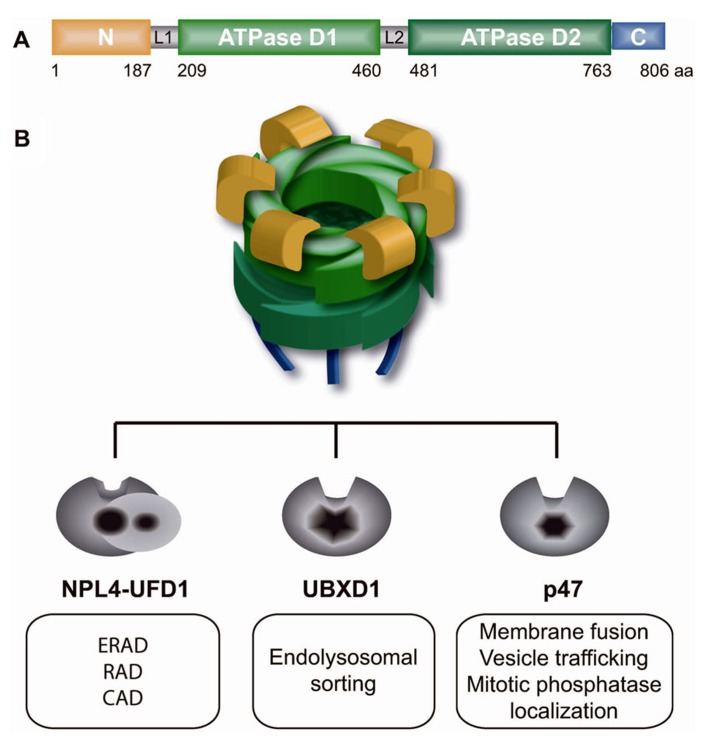
**p97 and its three core adaptors regulate diverse cellular functions.** **(A)** Domain structure of p97 monomer: N-terminal region (yellow), L1 and L2 linker regions (gray), two ATPase cassettes D1 and D2 (green), and the C-terminal tail (blue). **(B)** p97 homohexamer forms a barrel-like structure that mutually binds one of three core adaptor complexes, Npl4–Ufd1 heterodimer, UBXD1 or p47, and regulates diverse cellular functions as indicated in white boxes. ERAD, endoplasmic reticulum-associated degradation; RAD, ribosomal-associated degradation; CAD, chromatin-associated degradation.

Molecular chaperone p97, also known as VCP in vertebrates, Cdc48 in *S. cerevisiae*, Cdc-48 in *C. elegans*, TER94 in *Drosophila*, and VAT in archaebacteria, is a class II member of the ATPase associated with diverse cellular activities (AAA) ATPases (Woodman, 2003; Halawani and Latterich, 2006; Jentsch and Rumpf, 2007; Stolz et al., 2011; Meyer et al., 2012; Verma et al., 2013). p97 is highly conserved from archaebacteria to humans and is one of the most abundant proteins in the cytoplasm and nucleoplasm (Peters et al., 1990). It is a homohexameric barrel-like molecular machine composed of the following domains: N-terminal, two ATPase cassettes (D1 and D2) and a C-terminal domain (**Figure 1A**). The N-terminal region of p97 binds a myriad of substrates primarily embedded in different cellular structures, such as the endoplasmic reticulum (ER), ribosomes, membrane vesicles, spindles, or chromosomes. After it is bound to the substrate, p97 uses ATP hydrolysis in the D2 domain to stimulate major intrinsic conformational changes that are transmitted throughout the entire p97 molecule to the N-terminal domain (Rouiller et al., 2002; Li et al., 2012). Intrinsic conformational changes allow p97 to remodel and thus extract (segregase activity) bound substrates from diverse cellular structures or protein complexes. Beside its segregase activity from different cellular structures, p97 serves as unfoldase in processing of highly folded soluble proteasome substrates (Beskow et al., 2009). The majority of p97 substrates are ubiquitinated, and ubiquitin serves as a major regulator of p97 function (Ye, 2006). Identification of *Ufd1*, one of the core p97 adapters, and *Ufd2*, a p97-associated E4-ubiquitin ligase, as essential yeast genes in degradation of soluble substrate by ubiquitin fusion degradation (UFD) pathway was the first report of p97/Cdc48 function in the UPS (Johnson et al., 1995). p97 acts as a segregase or a molecular corkscrew for ubiquitinated substrates. Similar to ubiquitin and the UPS, p97 is involved in diverse, unrelated cellular functions and pathways. Interestingly, the specificity of p97 in diverse pathways and substrates is governed by different p97 adaptor proteins (also known as p97 cofactors) that typically possess ubiquitin-binding domains (Yeung et al., 2008; Hanzelmann et al., 2011; Kloppsteck et al., 2012). In general, p97 adaptors are only those proteins with p97-interacting domains or motifs, such as the UBX (ubiquitin regulatory X) domain, UBD (ubiquitin D) domain, PUB [PNGase (peptide *N*-glycosidase)/ubiquitin-associated] domain, SHP motif, VIP (VCP interacting protein) motif, or VBM (VCP-binding motif). UBX is the most prominent p97 interacting domain and has a similar structure to ubiquitin, but lacks sequence homology. There are at least 13 different UBX domain-containing proteins (UBX proteins) in mammalian cells, and their functions are largely uncharacterized. All UBX proteins were recently shown to interact with p97 as major regulators of p97 function (Alexandru et al., 2008). It is not known how many different p97 adaptor complexes exist in the cell, but current understanding of p97 and its adaptors suggests the existence of the following three primary mutually exclusive p97 core adaptor complexes: p97–Ufd1–Npl4 (p97^–Udf1^^–Npl4^), p97–p47 (p97^–p47^) and p97–UBXD1 (p97^–UBXD1;^ Meyer et al., 2012; **Figure 1B**). Each of the p97 adaptor complexes is dedicated to a specific ubiquitin-dependent cellular process. According to the “p97 core adaptor hypothesis,” the p97^–Ufd1^^–Npl4^ complex is involved in regulating mostly Lys48-polyubiquitinated substrates and proteasomal degradation in diverse cellular processes, such as ER-associated degradation (ERAD), chromatin-associated protein degradation (CAD), or ribosome-associated degradation (RAD). In contrast, the p97 core complexes p97^–p47^ and p97^–UBXD1^ are involved in ubiquitin-dependent proteasome-independent membrane fusion and vesicular trafficking processes. The specificity and activity of p97 core adaptor complexes to different substrates relies on a combination of secondary p97 adaptors in a process known as the p97 adaptor hierarchy (Hanzelmann et al., 2011). This is best illustrated in the p97^–Npl4^^–Ufd1^ complex, which processes and remodels various polyubiquitinated substrates involved in diverse pathways on the same cellular structure, such as chromatin (Alexandru et al., 2008; Verma et al., 2011; Davis et al., 2012; Mosbech et al., 2012). For example, the p97^–Ufd1^^–Npl4^ core complex is (i) recruited to stalled transcription by UBX4 and UBX5 (human homologs UBXD9 and UBXD7) and extracts RNA pol II from chromatin or (ii) recruited to stalled DNA replication by DNA damage-associated VCP/p97 cofactor 1 (DVC1; also known as SPARTAN or C1orf124) and extracts DNA pol delta and eta (Davis et al., 2012; Ghosal et al., 2012; Mosbech et al., 2012) or (iii) binds soluble hypoxia inducible factor 1a by UBXD7 (Alexandru et al., 2008). All of the proteins extracted, remodeled or unfolded due to p97 segregase and/or unfoldase activities are ultimately degraded by a proteasome.

In addition to its function in the UPS, in the last few years p97 function emerged in maintenance of cellular homeostasis by regulating two closely related processes, autophagy and endosomal trafficking (Ju et al., 2009; Tresse et al., 2010; Ritz et al., 2011). p97 plays an essential role in maturation of ubiquitin-coating autophagosomes, suggesting its function in autophagic degradation of ubiquitinated substrates. The focus of this review is the function of p97 in CAD and genome stability. For details on p97 function in autophagy and endosomal trafficking, see elsewhere (Ju and Weihl, 2010b; Bug and Meyer, 2012).

## CHROMATIN-ASSOCIATED PROTEIN DEGRADATION AND PROTEIN-INDUCED CHROMATIN STRESS

**FIGURE 2:**
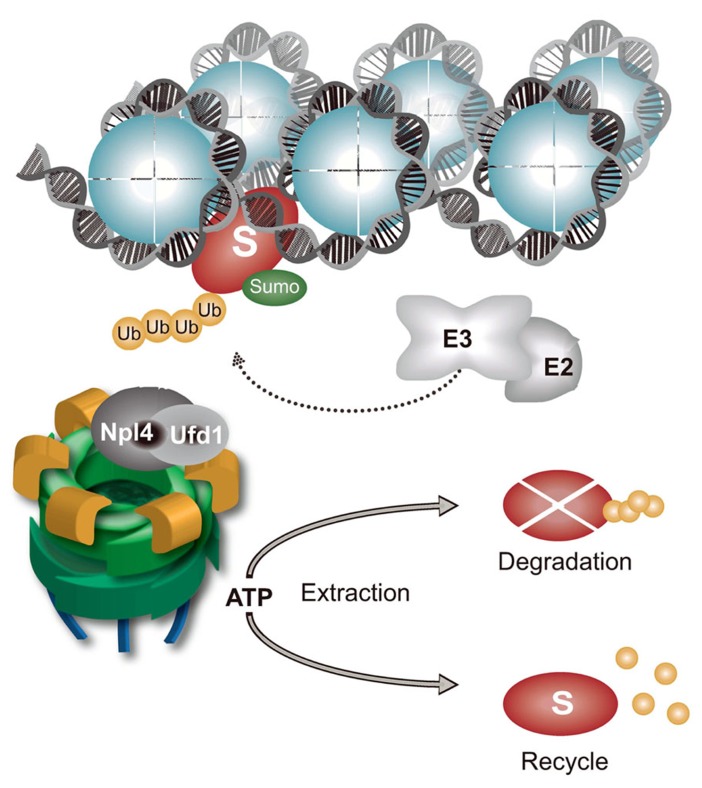
**Chromatin-associated protein degradation (CAD) regulated by p97^–Ufd1^^–Npl4^.** Chromatin-bound proteins (S represents any known p97 substrate on chromatin) are polyubiquitinated and sumoylated by specific E3 ubiquitin and SUMO ligases. p97^–Ufd1^^–Npl4^ complex is recruited to polyubiquitinated substrates by a ubiquitin-binding domain in Npl4 or Ufd1. ATP hydrolysis, located in the D2 ATPase cassette, induces conformational changes in the p97 molecule to remodel and release chromatin-bound substrates. Extracted substrates are either degraded by proteasomes or recycled. The p97^–Ufd1^^–Npl4^ complex maintains CAD and prevents PICHROS.

**FIGURE 3:**
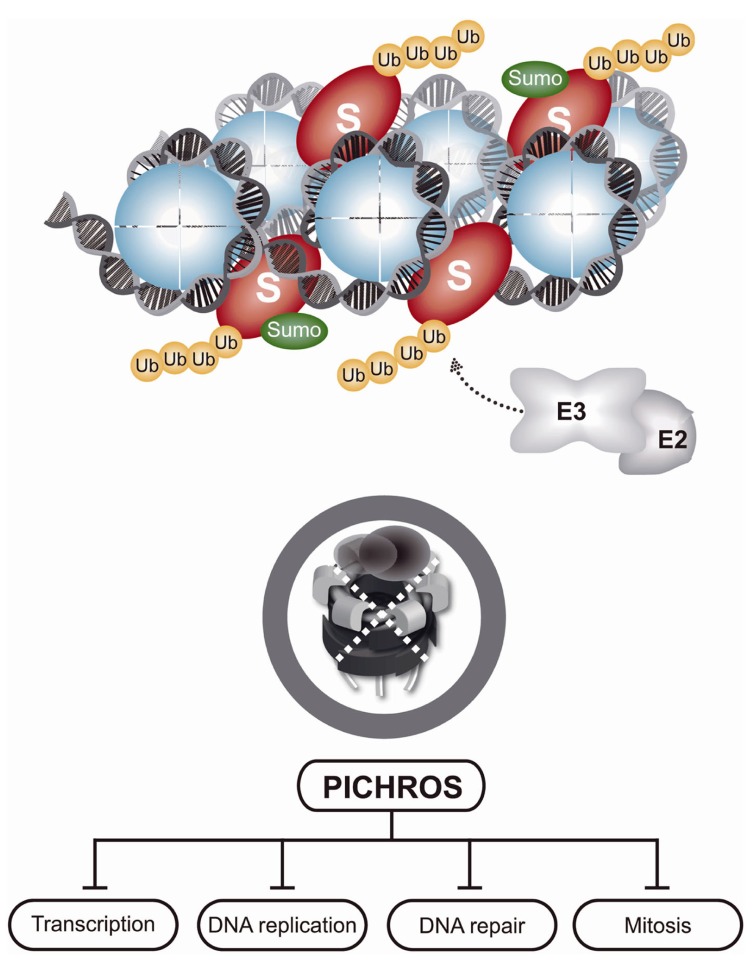
**Protein-induced chromatin stress (PICHROS).** As indicated in **Figure 2**, chromatin-bound substrates are polyubiquitinated, but cannot be removed due to inactivation of p97. Accumulation of polyubiquitinated substrates on chromatin causes PICHROS that severely disturbs, mostly inhibits, essential DNA metabolic processes, such as DNA replication, transcription, or DNA repair, and leads to genome instability.

Chromatin is a large protein-integration platform in which structural, dynamic, and spatiotemporal association with proteins involved in diverse processes, such as transcription, DNA replication, DNA repair, and cell division, must be tightly regulated. Nearly every protein recruited to chromatin during these processes that are essential for genome maintenance require timely removal or disassembly. Various components of the UPS and proteasome are tightly associated with chromatin, particularly when an increased protein turnover rate is needed, as in cases of DNA damage (Levy-Barda et al., 2011; Ramadan and Meerang, 2011; Butler et al., 2012). CAD plays an essential role in maintaining genome integrity and cellular homeostasis (**Figure 2**). Although chromatin is a key site of protein turnover necessary for genome stability, CAD has recently emerged as an important component of chromatin metabolism (Acs et al., 2011; Levy-Barda et al., 2011; Meerang et al., 2011; Feng and Chen, 2012; Galanty et al., 2012; Gudjonsson et al., 2012; Mallette et al., 2012; Mattiroli et al., 2012; Ramadan, 2012; Yin et al., 2012). Many proteins that operate on chromatin are tightly bound to the chromatin structure or chromatin-associated processes and are even considered insoluble. In addition to the physical presence of proteasomes in the vicinity of chromatin, mechanical force is needed to remodel and disassemble chromatin-bound substrates for final removal and degradation. Discovery of the ubiquitin-dependent molecular segregase p97 as an integral part of CAD sheds new light on how cells disassemble proteins from chromatin (Ramadan, 2012). Inactivation of p97 by RNAi or p97 segregase mutants in various systems (e.g., yeast and humans) causes K48-polyubiquitinated p97 substrates to accumulate on chromatin, leading to PICHROS. PICHROS negatively affects downstream events that involve accumulated substrates, such as DNA replication, DNA repair, mitosis, and transcription (**Figure 3**; Ramadan et al., 2007; Franz et al., 2011; Meerang et al., 2011; Raman et al., 2011; Verma et al., 2011). To our knowledge, the first identified chromatin-associated substrate of p97 was Aurora B (Ramadan et al., 2007). Since this discovery, information regarding removal of chromatin substrates by ubiquitin-dependent p97 activity has rapidly progressed (Wilcox and Laney, 2009; Acs et al., 2011; Franz et al., 2011; Meerang et al., 2011; Raman et al., 2011; Davis et al., 2012; Ghosal et al., 2012; Mosbech et al., 2012). The importance of CAD function in genome stability was further confirmed by discovery of K48 ubiquitinated substrates orchestrated by E3 ubiquitin ligase RNF8 and p97 at sites of DNA damage (Meerang et al., 2011). Understanding the function of CAD in genome stability has rapidly progressed (see above). Tight collaboration between ubiquitination and sumoylation at sites of DNA damage plays a role in CAD. The evolutionary conserved SUMO-targeted E3 ubiquitin ligase (STUbL) RNF4 in mammals, Slx5–Slx8 in budding yeast and Rfp1/2–Slx8 in fission yeast is recruited to sumoylated substrates at sites of DNA damage (Prudden et al., 2007; Galanty et al., 2012; Yin et al., 2012). STUbL polyubiquitinates SUMO-modified proteins and primes them for proteasomal degradation. Inactivation of STUbL causes hyperaccumulation of SUMO conjugates and SUMO-induced cell toxicity. In human cell lines, RNF4 is recruited to sumoylated substrates and polyubiquitinates DSB-induced factors, such as mediator of DNA damage checkpoint 1 (MDC1) and replication factor A (RPA). Consequently, polyubiquitinated MDC1 and RPA are removed from sites of damage in a proteasome-dependent manner (Shi et al., 2008; Galanty et al., 2012). RNF4-dependent MDC1 and RPA turnover allow recruitment of downstream factors, such as BRCA2 and Rad51, essential for efficient DNA repair. Inactivation of RNF4 also causes persistence of other factors at sites of damage, such as E3 ubiquitin ligases RNF8 and RNF168 and DNA damage signaling factor 53BP1 (Yin et al., 2012). Two additional E3 ubiquitin ligases, UBR5 and TRIP12, are involved in CAD and protection from PICHROS (Gudjonsson et al., 2012). UBR5 and TRIP12 regulate turnover of RNF168, the primary E3 ubiquitin ligase that ubiquitinates histones after DSBs. Depletion of UBR5 and TRIP12 causes hyperaccumulation of RNF168 and a significant increase in ubiquitin conjugates, which stimulates widespread accumulation of ubiquitin-dependent factors 53BP1 and BRCA1. Deregulation of spatiotemporal SUMO- and ubiquitin-dependent protein turnover may cause hyperaccumulation of various proteins on chromatin, consequently leading to PICHROS and genome instability. In the following text, we will focus on p97-dependent CAD and PICHROS directly related to genome stability. We will also elaborate the function of p97 in cell cycle, which may indirectly affect genome stability. 

## p97 IN THE CELL CYCLE

Cell cycle progression can be simplified into two main processes, DNA replication (S phase) and segregation of replicated chromosomes into two daughter cells (M phase). The G1 and G2 phases are intercalated to ensure that all requirements necessary for safe DNA replication and segregation are achieved. The transition from one cell cycle phase to another is controlled by numerous mechanisms to ensure correct cell division. Cyclin-dependent kinases (CDKs) play a central role in cell cycle progression. The activity of CDKs is coordinated by transcription and ubiquitin-dependent degradation of different cell cycle-specific cyclins, CDK inhibitors, and phosphatases (Malumbres and Barbacid, 2009). Additionally, checkpoint mechanisms activated upon DNA damage ensure the quality of DNA during replication and segregation (Branzei and Foiani, 2009).

The *Cdc48* gene (p97 homolog) was identified in the first genetic screen for cell division cycle (cdc) mutants in yeast (Moir et al., 1982). Cdc48 generally attaches to the ER, but relocalizes in the nucleus after phosphorylation in a cell cycle-dependent manner (Madeo et al., 1998). Several observations in yeast and other organisms have revealed that Cdc48 is crucial for normal cell cycle progression and associated genomic stability (Mouysset et al., 2008; Deichsel et al., 2009). In yeast, mutations in the *Cdc48* gene cause delayed G1/S transition and G2/M arrest. In *C. elegans* embryos, depletion of Cdc-48 or one of its cofactors, Ufd-1 or Npl-4, causes delays in S phase progression due to activation of replication checkpoints (Mouysset et al., 2008). p97 or Ufd1–Npl4 inactivation also leads to delayed progression through anaphase and exit from mitosis in human cell lines and *Xenopus* egg extracts (Ramadan et al., 2007; Dobrynin et al., 2011). These p97-defective phenotypes clearly demonstrate the crucial function of p97 in different phases of the cell cycle. p97 is important for protein degradation, and cell cycle progression requires removal/degradation of cell cycle related proteins. Determining the role of p97 in regulating these processes is important for understanding how protein recycle/degradation affects the normal cell cycle.

### p97 IN G1/S TRANSITION

Many cell fate decisions are determined in the G1 phase of the cell cycle. A key question is whether or not to proliferate (replicate). When the cellular environment is favorable, cells initiate the division cycle. The cellular commitment to replicate the genome and divide is known as the restriction point. After passing the restriction point, cells switch from mitogen-dependent growth in early G1 to growth factor-independent progression in S phase, which is controlled by the retinoblastoma protein (pRb) and the cdk4/cyclin D complex. Cdk4/cyclin D hyperphosphorylates pRb, which consequently releases E2F transcription factors that activate transcription of several regulatory genes necessary for G1/S transition and S phase progression, such as cyclin E and cyclin A. The activity of cdk4/cyclin D needs to be tightly regulated (Malumbres and Barbacid, 2009).

Mutations in the *Cdc48* gene delay G1/S transition in budding yeast (Fu et al., 2003). Cdc28/Cln, a yeast Cdk1/cyclin important for G1/S transition controls the execution of Start (a yeast cell cycle commitment point equivalent to the restriction point in mammalian cells). Far1p is a Cdc28/Cln inhibitor and its degradation is needed for G1/S progression. Cdc48 physically interacts with ubiquitinated Far1p and stimulates its degradation. The defect in G1/S transition after Cdc48 inactivation was shown to be due to persistence of the Cdc28/Cln inhibitor Far1p. In contrast to normal cells in which Far1p is degraded following release from G1 arrest, *Cdc48* mutant cells accumulated ubiquitinated Far1p. G1/S delay could be rescued following mutations in both *Cdc48* and *Far1p* genes, clearly suggesting that Cdc48 is required for Far1p degradation. Although no data are available, a similar p97-dependent process could exist in higher eukaryotes in which CDK activity is also regulated by CDK inhibitors. In addition to Far1p degradation, Cdc48^–Ufd1^^–Npl4^ complex controls G1/S transition via cell wall integrity pathway mechanisms in yeast (Hsieh and Chen, 2011). The mechanisms by which Cdc48 controls cell wall integrity have not been determined, although Cdc48 appears to regulate Mpk1 activity, which is a MAP kinase family member important for cell wall integrity, in response to stress conditions, including heat shock.

### p97 IN DNA REPLICATION AND S PHASE

To divide and preserve an intact genome, cells must tightly regulate DNA replication. DNA synthesis occurs in the S phase, but preparation starts in late mitosis and G1 by loading a pre-replicative complex (pre-RC) at each origin of replication. Some pre-RCs are active, while others remain dormant. Pre-RCs consist of an origin recognition complex (ORC), cell division control protein (Cdc6), chromatin licensing and replication factor (Cdt1), and the minichromosome maintenance (MCM) helicase complex. Pre-RC is activated by phosphorylation to recruit essential replication factors, such as MCM-10, CDC-45, and the Go–Ichi–Ni–San complex (GINS), to form a pre-initiation complex (pre-IC), which recruits DNA primase and polymerases to initiate DNA synthesis. After the origins fire, the pre-RC factors are removed or inhibited to prevent re-replication of the genome during the same cell cycle. Various obstacles during DNA synthesis, such as secondary DNA structures, DNA–protein complexes or damaged bases, can stall the fork or lead to fork collapse. To ensure that the DNA replication fork can handle all of these challenges, cells activate an intra-S phase checkpoint (Branzei and Foiani, 2010).

p97 function includes regulation of DNA replication and progression through the S phase (Mouysset et al., 2008). Depletion of Cdc-48 (p97) or its adaptors Ufd-1 or Npl-4 in *C. elegans* significantly delays progression through the S phase due to activation of atl1 (ATR) and the Chk1-dependent intra-S phase checkpoint. Depletion of Cdc-48, Ufd-1, or Npl-4 reduces the number of nuclei and total amount of DNA and increases the number of chromosomal bridges in *C. elegans* embryos. Depletion of intra-S phase checkpoint kinases completely restores the delay in S phase progression in Cdc-48-, Npl-4-, or Ufd-1-defective cells, but could not restore the number of nuclei, DNA content or decrease the number of chromosomal bridges. Recent work by two independent groups provided mechanistic insight into the role of p97^–Ufd1^^–Npl4^ in DNA replication (Franz et al., 2011; Raman et al., 2011). The research groups showed that p97 regulates Cdt1 chromatin turnover and stability via two distinct pathways, (i) a UV lesion-related pathway (Raman et al., 2011) and (ii) firing and elongation of the replication fork (Franz et al., 2011). In the UV lesion-related pathway, p97 regulates destruction of Cdt1 at sites of UV-induced DNA damage. After UV damage, nucleotide excision repair (NER) machinery recognizes distorted DNA (thymine dimers) and excises damaged DNA strands 25–30 nucleotides in length. This gap is repaired by DNA polymerization in proliferating cell nuclear antigen (PCNA)- and E3 ubiquitin ligase Cul4–DDB2-dependent manners. Cdt1 associates with PCNA to initiate DNA replication at sites of damage, but must be tightly regulated to prevent re-replication. After initiation of DNA synthesis, PCNA-bound Cdt1 is polyubiquitinated by Cul4–DDB2, extracted from chromatin in a p97^–Ufd1^^–Npl4^-dependent manner and presented to the proteasome for final degradation (Raman et al., 2011). p97 directly controls gap-filling DNA synthesis after UV damage. In the second pathway, p97 controls DNA replication by extracting polyubiquitinated Cdt1 from chromatin under physiological conditions in *C. elegans* and a *Xenopus* egg extract. Inactivation of the p97^–Ufd1^^–Npl4^ complex stabilizes Cdt1 on interphase and mitotic chromatin. Consequently, Cdc45 and GINS are stabilized on chromatin-bound Cdt1 and could interfere with DNA replication fork elongation (Franz et al., 2011). Cdc-48/p97 depletion leads to accumulation of Cdt1 on chromatin in *C. elegans* embryos, *Xenopus* egg extract and human cell lines. Re-replication, a typical phenotype caused by Cdt1 overexpression, was not observed in p97/Cdc-48 depleted cells. In contrast, two independent research studies showed that inactivation of p97 caused G2/M arrest in human embryonic kidney cell line (HEK293) and decreased the total DNA content in *C. elegans* (Mouysset et al., 2008; Raman et al., 2011). This is diametrically different from a typical re-replication phenotype, which involves an increased amount of total DNA characterized by elevated Cdt1 protein levels (Teer and Dutta, 2008; Ramanathan and Ye, 2011). The molecular mechanisms involving p97 regulation of DNA replication cannot be simply attributed to extraction and stability of Cdt1 from chromatin. However, the evolutionarily conserved function of p97^–Ufd1^^–Npl4^ in DNA replication has been demonstrated (Mouysset et al., 2008; Deichsel et al., 2009; Franz et al., 2011; Raman et al., 2011). p97 most likely regulates multiple steps during DNA synthesis. This hypothesis could be further supported by direct physical interactions with several replicative helicases, such as the Werner protein and HIM-6 (Bloom helicase homolog; Partridge et al., 2003; Indig et al., 2004; Caruso et al., 2008).

### p97 IN MITOSIS

Mitosis is characterized by profound changes in cell physiology that allow nuclear envelope disassembly and separation of genetic material into two daughter cells. Spatiotemporal coordination of complex mitotic processes depends on phosphorylation and ubiquitination events that are crucial for genome integrity. At the end of mitosis, inhibition/degradation of several kinases, such as cyclin B and Aurora B, leads to spindle disassembly, cytokinesis, chromatin decondensation, and nuclear envelope reformation (Carmena et al., 2012).

Aurora B was the first p97 substrate discovered in chromatin (Ramadan et al., 2007). In *Xenopus* egg extracts and *C. elegans*, p97/Cdc-48^–Ufd1^^–Npl4^ binds polyubiquitinated Aurora B and extracts it from mitotic chromatin. p97-dependent removal of Aurora B allows chromatin decondensation and nuclear envelope formation during exit from mitosis. A similar mechanism was observed in human cell lines, although regulation of Aurora B activity by p97 initiates much earlier during mitosis (Dobrynin et al., 2011). Depletion of Ufd1 or Npl4 causes an increase in Aurora B activity, which leads to defects in chromosomal alignment in anaphase, resulting in missegregated chromosomes and multi-lobed nuclei. These results establish p97^–Ufd1^^–Npl4^ as a negative regulator of Aurora B activity, which regulates multistep processes in chromosome dynamics during mitosis.

Similarly, Cdc-48 in *C. elegans* was shown to be required for proper condensation and segregation of meiotic chromosomes by controlling AIR-2/Aurora B (Sasagawa et al., 2012). Cdc-48 is required for localization of AIR-2 at regions between homologous chromosomes in meiosis I. In the absence of Cdc-48, higher levels of AIR-2 increase phosphorylation of its substrates over the entire length of the chromosomes, leading to defective chromosome segregation.

In contrast to the p97^–Ufd1^^–Npl4^ core complex, which extracts and inactivates Aurora B in mitosis, another p97 core complex (p97^–p47^) balances Aurora B activity in mitosis using a different mechanism (Cheng and Chen, 2010). The Cdc-48^–Shp1^ complex (p97^-p47^ in metazoans) facilitates nuclear localization of Glc7, the yeast ortholog of protein phosphatase-1 (PP1), which counteracts Aurora B kinase activity. Inactivation of the Cdc-48^–Shp1^ complex causes cell cycle arrest in metaphase due to a defect in bipolar attachment of the kinetochore that activates the spindle checkpoint.

In addition to Aurora B regulation, Cdc48/p97 is required for proper spindle disassembly at the end of mitosis (Cao et al., 2003). *Xenopus* egg extracts containing the dominant negative form of p97 (p97QQ) or depleted of Ufd1 or Npl4 cofactors were unable to disassemble the spindle and reform interphase microtubules (MTs) and remained in a mitotic state. Cdc48/p97^–Ufd1^^–Npl4^ specifically interacts with spindle assembly factors XMAP215, TPX2, and Plx1 at the exit of mitosis, promoting its sequestration in the cytoplasm or extraction from MTs. This function of p97 was further confirmed in yeast where p97 (Cdc48) is required for degradation of spindle assembly factors Ase1 and Cdc5.

In conclusion, two p97 core complexes, p97^–Npl4^^–Ufd1^ and p97^–p47^, coordinate Aurora B activity in chromatin to allow proper chromosomal alignment, segregation, decondensation, and nuclear envelope formation at mitotic exit. In addition to direct effects on mitotic chromatin, the p97^–Ufd1^^–Npl4^ complex also regulates spindle disassembly at the end of mitosis.

## p97 IN DNA DAMAGE

To cope with DNA damage, preserve genetic information for the next generation and survive, cells have evolved a variety of DNA repair mechanisms specific for different types of damage, which are orchestrated by DDR and DDT (Nyberg et al., 2002; Jackson and Bartek, 2009; Curtin, 2012). Although p97 phosphorylation after IR and physical interaction with BRCA1 predicted the role of p97 in DNA repair more than a decade ago, its function in DNA repair remained a mystery (Zhang et al., 2000; Livingstone et al., 2005). The molecular mechanism of p97 emerged in DSB repair, TLS and transcription-coupled NER (TC-NER) only recently (Acs et al., 2011; Meerang et al., 2011; Verma et al., 2011; Davis et al., 2012; Ghosal et al., 2012; Mosbech et al., 2012). Whether p97 is involved in other DNA repair pathways remains to be discovered.

### DVC1 LINKS p97 TO TRANSLESION DNA SYNTHESIS

DNA is particularly vulnerable to damage during DNA replication. Some base adducts may escape detection by repair proteins before progression through the S phase. During replication, these lesions cannot be accommodated at the active sites of replicative DNA pols and stall progression of the DNA replication fork (Lehmann, 2011a). Stalled replication forks activate DDT pathways and recruits translesional DNA pols, a mechanism known as TLS (Ulrich, 2007; Sale et al., 2012). TLS uses a DNA pol switch mechanism, modulated by the ubiquitination status of PCNA, in which replicative DNA pols are exchanged for translesion DNA pols (Lehmann, 2011b).

Translesion DNA synthesis is initiated by monoubiquitination of PCNA by the E3 ubiquitin ligase Rad18. Specialized translesion DNA pols are recruited to monoubiquitinated PCNA at stalled replication forks to promote bypass of damaged DNA. However, replication through a lesion site often requires the sequential action of two DNA pols in which one inserts a nucleotide opposite the damage (DNA pol eta) and the other extends from the inserted nucleotide (DNA pol zeta; Lange et al., 2011). After lesion bypass, PCNA is deubiquitinated by ubiquitin protease USP1, which stimulates the switch from translesion to replicative polymerases and continuation of DNA replication (Fox et al., 2011).

A newly identified p97 adaptor, DVC1, also known as Spartan, recently emerged as a central factor in TLS (Centore et al., 2012; Davis et al., 2012; Ghosal et al., 2012; Juhasz et al., 2012; Machida et al., 2012; Mosbech et al., 2012; Kim et al., 2013). DVC1 is conserved from *C. elegans* to humans and it localizes to UV-induced DNA lesions and other DNA replication-related damage, but not to DSBs induced by IR. DVC1 depleted cells were shown to be sensitive to various replication-related genotoxic agents. Together with physical interaction and cellular colocalization with PCNA, these findings emphasize the role of DVC1 in replication-related DNA damage processes, but not DSB repair after IR.

DVC1 contains several domains, such as a putative zinc metalloprotease domain (SprT), a p97-interacting motif (SHP), a PCNA interaction domain (PIP), and a zinc finger ubiquitin-binding domain (UBZ4), that link its function to ubiquitin-dependent and p97-regulated replication-related processes (Davis et al., 2012; Ghosal et al., 2012; Mosbech et al., 2012). In addition to interacting with PCNA, DVC1 interacts with other essential proteins involved in DNA replication, TLS, and UPS, such as DNA pol delta, eta, Rad18, ubiquitin, and the p97^–Ufd1^^–Npl4^ complex (Centore et al., 2012; Davis et al., 2012; Ghosal et al., 2012; Machida et al., 2012; Mosbech et al., 2012; Kim et al., 2013). DVC1 was shown to directly interact with p97 at the SHP domain, which is essential for recruiting p97 to stalled replication forks. DVC1 lacking the SHP domain can colocalize with DNA lesions, but cannot recruit p97. These data suggest undisputable roles of DVC1 and p97 in TLS synthesis.

However, the exact mechanism of DVC1 function and its collaboration with p97 at stalled replication forks are still not completely understood. First, it is not clear whether recruitment of DVC1 to stalled replication forks depends on Rad18 E3 ligase. Although three research groups claim that DVC1 recruitment to UV lesions is dependent on Rad18 (Centore et al., 2012; Ghosal et al., 2012; Juhasz et al., 2012), two other research groups have reported that DVC1 recruitment does not depend on Rad18 (Davis et al., 2012; Mosbech et al., 2012). Second, two groups claim that DVC1 brings Rad18 to chromatin and enhances monoubiquitination of PCNA (Centore et al., 2012; Ghosal et al., 2012), while others did not observe this effect. Third, one research group also suggested that DVC1 is recruited to stalled replication forks by Rad18 and PCNA-monoubiquitination, protecting monoubiquitinated PCNA from deubiquitination (Juhasz et al., 2012). Fourth, two groups have reported that DVC1 recruits p97 to stalled replication forks to extract DNA pol eta and allows the switch to replicative polymerases after bypassing the UV lesion (Davis et al., 2012; Mosbech et al., 2012). In contrast, one group showed that DVC1 has an opposite function by removing replicative polymerase delta at UV lesions to allow recruitment of DNA pol eta (Ghosal et al., 2012).

In addition to above-mentioned DVC1 function in error-free TLS orchestrated by the switch between DNA pol eta and DNA pol delta, DVC1 also negatively influences error-prone TLS orchestrated by a DNA pol eta/DNA pol zeta switch supported by Rev1 and PolD3 (Kim et al., 2013). DVC1 and its SprT domain suppress PolD3 from binding to DNA pol zeta to inhibit error-prone TLS.

Even thought the exact mechanistic insight of DVC1 in TLS is not yet clear, DVC1 plays a crucial role in TLS, likely by stimulating error-free TLS and inhibiting error-prone TLS. Further research will be needed to elucidate the exact function of DVC1 at stalled replication forks and involvement of p97.

### p97 IN DNA DOUBLE-STRAND BREAK REPAIR AND DAMAGE RESPONSE

Double-strand break is the most deleterious DNA lesion. If not repaired, it can cause chromosomal rearrangements, deletions and genome instability or cell death. A single DSB can cause cell lethality in yeast, suggesting that it is a highly cytotoxic DNA lesion that must be immediately detected and processed (Bennett et al., 1993). It is estimated that approximately 1% of endogenous single-strand DNA breaks convert to DSBs during S phase in mammalian cells, which correlates to about 50 endogenous DSBs per cell cycle (Vilenchik and Knudson, 2003). DSBs also occur as a consequence of certain medical interventions related to diagnostics or therapy (e.g., X-ray). To prevent genome instability, ubiquitination and sumoylation have emerged as an essential PTM in regulating DDR and DSB repair.

Until recently, the RNF8/RNF168-dependent Lys63-ubiquitin chain was regarded as the sole ubiquitin-signaling pathway at sites of DSB (Doil et al., 2009; Stewart et al., 2009; Al-Hakim et al., 2010; Ulrich and Walden, 2010; Mattiroli et al., 2012). This paradigm changed when p97 function was identified in DDR and DSB repair (Acs et al., 2011; Meerang et al., 2011; Ramadan, 2012). The p97^Ufd1^^–Npl4^ complex is recruited to sites of DSB soon after damage occurs. Recruitment of the p97^Ufd1^^–Npl4^ complex to sites of DSBs strongly depends on free nuclear ubiquitin and RNF8 E3-ubiquitin ligase activity, but not E3 ligase RNF168 (Meerang et al., 2011). This suggests the presence of an RNF8/RNF168-orchestrated cascade and a sole RNF8-orchestrated cascade at DNB sites, which was confirmed using ubiquitin chain-specific Lys63 or Lys48 antibodies. While depletion of RNF8 and RNF168 completely abolished formation of the Lys63- and Lys48-ubiquitin chains at sites of DNA damage, depletion of RNF168 only eliminated Lys63-ubiquitin chain formation. Recruitment of p97 to DSB sites strongly depends on RNF8/K48-ubiquitin chain formation. Depletion of p97 or expression of p97 segregase mutants caused increased levels and persistence of Lys48-ubiquitin chains at DSB sites. These data clearly demonstrate that p97 is recruited to DSB sites via Lys48-ubiquitin chains formed by RNF8 and extracts them from chromatin. p97 processing of Lys48-ubiquitinated substrates soon after DSBs is important for recruiting signaling and repair molecules, such as 53BP1, BRCA1, and Rad51 (Meerang et al., 2011). The inability of p97-inactivated cells to process Lys48-ubiquitinated substrates at sites of DSBs severely reduces the main branches of DSB repair, homologous recombination, and non-homologous end-joining. The specific p97 substrates at sites of DNA damage and mechanisms by which p97 influences the main branches of the DSB repair pathway remain unknown. Discovery of the p97 substrate polycomb protein L3MBTL1 in the vicinity of DSBs explains why inactivation of p97 segregase activity eliminated 53BP1 protein recruitment. L3MBTL1 possesses multiple tandem Tudor domains, which enable its high affinity to bind H4Lys20me2 in undamaged chromatin (Acs et al., 2011). After DSBs occur, L3MBTL1 is removed by p97 and allows recruitment of 53BP1 by its own Tudor domain to H4Lys20me2. p97 regulation in recruiting two other essential signaling and repair proteins, BRCA1 and Rad51, remains to be investigated. These data establish previously unrecognized RNF8-Lys48 ubiquitin cascades at DSB sites, which recruit p97^–Ufd1^^–Npl4^ segregase activity to process Lys48-ubiquitin substrates (Ramadan, 2012).

Cdc48^–Ufd1^^–Npl4^, a yeast homolog of p97^Ufd1^^–Npl4^, is involved in processing sumoylated and ubiquitinated substrates at sites of DNA damage (Nie et al., 2012). Yeast Ufd1 contains a SUMO-interacting motif (SIM) that non-covalently binds SUMO conjugates and recruits the Cdc48^–Ufd1^^–Npl4^ complex to STUbL targets. Cdc48^–Ufd1^^–Npl4^ cooperates with STUbL in DNA repair to reduce stalled covalent DNA topoisomerase1-DNA adducts by binding and processing sumoylated and ubiquitinated substrates at sites of DNA damage. Whether the human p97^–Ufd1^^–Npl4^ complex also participates in dual recognition of sumoylated and ubiquitinated substrates at sites of DNA damage is not known.

### p97 IN TRANSCRIPTION-COUPLED NUCLEOTIDE EXCISION REPAIR

A wide variety of DNA helix-distorting lesions, such as UV-induced photolesions (thymine dimers) and DNA adducts induced by chemicals in transcribed DNA, block progression of the RNA pol II complex. Blocking transcription at sites of DNA damage represents a serious obstacle for DNA replication and leads to DNA replication fork collapse. To prevent DNA replication fork collapse at sites of stalled transcription and avoid apoptosis, cells activate TC-NER, a subpathway of NER. TC-NER rapidly removes lesions from the transcribed strand and allows transcription to continue (Fousteri and Mullenders, 2008; Lehmann, 2011a). Upon induction of DNA damage by UV or UV-mimetic drugs, thymine dimers form, transcription stalls, and the TC-NER subpathway is activated. Stalled transcription recruits E3 ubiquitin ligase Cul3 to ubiquitinate the RNA pol II complex and prime it for proteasomal degradation (Chen et al., 2007; Ribar et al., 2007). It is hypothesized that the stalled RNA pol II complex shields DNA lesions and prevents access by the NER machinery. As a result, the complex must be removed and degraded. The Cdc48^Ufd1^^–Npl4^ complex together with Ubx4 and Ubx5 (human homologous UBXD9 and UBXD7) play an important role in UV-dependent turnover of the stalled RNA pol II complex in yeast (Verma et al., 2011). The Cdc48^Ufd1^^–Npl4^^–Ubx4^^–Ubx5^ complex is recruited at sites of UV-induced damage and facilitates CAD of Rpb1, the largest RNA pol II subunit. The stalled RNA pol II complex also stimulates increased proteasome recruitment at sites of UV lesions that form a tight complex with p97/Cdc48. These results suggest tight cooperation between p97/Cdc48 and proteasomes at sites of UV lesions, in the earliest step of TC-NER. Beside its involvement in the upstream step of TC-NER, p97 also operates in the gap-filling DNA synthesis, which is the final step of both NER subpathways, TC-NER and global genome-NER (GG-NER; Raman et al., 2011). During gap synthesis, p97 promotes segregation and degradation of PCNA-bound Cdt1 to prevent re-replication (Raman et al., 2011; Ramanathan and Ye, 2011). Together, these results suggest that p97 operates at different levels in both NER subpathways. It would be interesting to see whether and how p97 operates in early steps of GG-NER.

## p97 AND DISEASES RELATED TO GENOME STABILITY

p97 has been implicated in the pathogenesis of many human diseases, including breast, colorectal, lung, prostate and pancreatic cancers, chronic obstructive pulmonary disease and severe emphysema, cystic fibrosis, Alpha-1-trypsin deficiency, Paget’s disease of bone, and several types of neurodegenerative disorders (Vij, 2008; Haines, 2010; Min et al., 2011). Inclusion body myopathy associated with Paget’s disease of bone and frontotemporal dementia (IBMPFD) is the only disorder that has been directly linked to p97 dysfunction to date (Kimonis et al., 2008). p97 mutations have been linked to 2% of isolated familial amyotrophic lateral sclerosis (ALS; Johnson et al., 2010). IBMPFD is an autosomal dominant negative inherited degenerative disorder due to a single missense mutation in p97 in humans. There are several families with IBMPFD worldwide, but the mutations are primarily located in the N-terminal region, the linker region between the N-terminal and D1 areas and the D1 region (Weihl et al., 2009). IBMPFD is characterized by disabling weakness, osteolytic lesions consistent with Paget’s disease of bone and frontotemporal dementia. Accumulation of cytoplasmic and nuclear ubiquitin-positive aggregates in the tissues of patients with IBMPFD suggests that p97 processing of ubiquitinated substrates is the key mechanism involved in pathogenesis. Because p97 processes substrates for lysosomal and proteasomal degradation pathways, it is not clear which pathway is affected in IBMPFD (Janiesch et al., 2007; Ju and Weihl, 2010a; Ritz et al., 2011).

IBMPFD patients do not suffer from genomic instability phenotypes characterized by premature aging and/or cancer development. For example, the incidence of osteosarcoma complications in Paget’s disease of bone is less than 1% (Hansen et al., 2006). In contrast, proteasomal degradation plays a crucial role in various aspects of genome stability (Shi et al., 2008; Ramadan and Meerang, 2011; Galanty et al., 2012; Ramadan, 2012). Defects in lysosomal degradation rather than defects in proteasomal degradation likely contribute to the pathogenesis of IBMPFD. There is evidence to support the hypothesis that defective p97 functioning in lysosomal degradation is the primary cause of IBMPFD.

Although there is no direct proof that p97 mutations play a role in diseases associated with genome stability, we provide evidence in this review to support an essential, conserved role of p97 in genome stability from yeast to humans. In addition, p97 or its adaptors regulate proteins involved in tumorigenesis, such as Aurora B (overexpressed in cancer cells and implicated in genome stability), IκB [potential inhibitor of the pro-survival function of nuclear factor kappa B (NFκB)] or HIF1a (promoter of tumor angiogenesis and metastases; Asai et al., 2002; Ramadan et al., 2007; Alexandru et al., 2008; Dobrynin et al., 2011). Elevated p97 expression correlates with the progression, prognosis, and metastatic potential of many cancers (Yamamoto et al., 2003, 2004a,b,d). For example, high levels of p97 protein correlate with colorectal carcinomas (Yamamoto et al., 2004c). Low p97 expression levels have been observed in adenomas, while high levels have been shown in all metastatic tumors. Patients with tumors that express high levels of p97 show a higher recurrence rate and poorer disease-free periods and overall survival compared to patients with tumors that have low p97 expression, suggesting that high levels of p97 indicate a poor prognosis. Similar to colorectal cancer, p97 is overexpressed in non-small cell lung carcinoma (NSCLC; Yamamoto et al., 2004b). All of the data described are based on a correlation between p97 protein levels and cancer development and progression. Whether elevated p97 expression increases the degradation of growth inhibitory proteins or elevated expression is a cellular response to protein-induced stress in cancer is not known (Haines, 2010). However, inhibition of p97 by a small molecule significantly reduced NSCLC tumor growth in *in vitro* and *in vivo* models (Valle et al., 2011). This suggests that increased p97 levels may be directly responsible for tumorigenesis.

There is no evidence that mutations in p97 are related to cancer. Considering its ubiquitin-dependent functions in diverse processes related to proliferation and maintenance of protein homeostasis, one could speculate that cancer cells rely on intact p97 function.

Because p97 is essential for cell survival, alterations in its adaptors would be expected to cause cancer. Haploinsufficiency of the FAF1 gene, a member of the UBX family of adaptors, was observed in 30% of cervical carcinomas and 12.5% of mantle-cell lymphomas (Hidalgo et al., 2005; Bea et al., 2009). FAF1 protein levels are downregulated in gastric carcinomas and a large percentage of mesotheliomas (Bjorling-Poulsen et al., 2003; Altomare et al., 2009).

p97 and the FAF1 adaptor likely play important roles in cancer development, although clear molecular mechanisms have not been elucidated.

Direct evidence of p97 involvement in genome stability and cancer development was recently emerged by the discovery of a novel premature aging syndrome due to a homozygotic mutation in the p97 adaptor DVC1. DVC1 recruits p97 to stalled replication forks and is essential in preventing mutagenesis. A DVC1 mutation in novel premature aging syndrome eliminated recruitment of p97 to stalled replication forks, which consequently induced DNA replication fork collapse and an increased level of chromosomal aberrations (our unpublished results, D. Lessel et al., submitted).

## CONCLUSION

**FIGURE 4:**
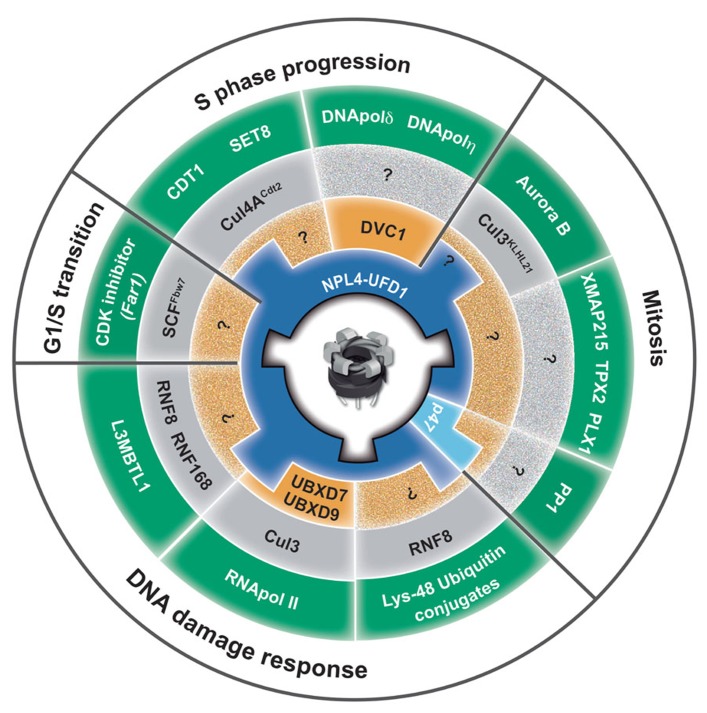
**Role of p97 in genome stability.** The circle represents a summary of p97 functions in diverse cellular processes, from yeast to human, essential for maintenance of genome stability. Key players from different species are abbreviated with human homologous (e.g., yeast Cdc48 and Ubx5 is equivalent to human p97 and UBXD7). The p97 homohexamer is centrally located. The first ring (blue) represents two core adaptor complexes, Ufd1–Npl4 and p47. The second ring (orange) represents the next tier of p97 adaptors that direct the function of p97 core complexes. DVC1 directs p97^–Ufd1^^–Npl4^ function toward TLS, and UBXD7 and UBXD9 direct p97^–Ufd1^^–Npl4^ toward stalled transcription and TC-NER. The third ring (gray) represents E3 ubiquitin ligases that ubiquitinate p97-substrates. The fourth ring (green) represents p97-substrates that must be remodeled (extracted) by p97 to avoid PICHROS and prevent genome instability. The fifth ring (white) represents different cellular processes in which p97 plays an essential role for maintenance of genome stability. PP1, protein phosphates 1/Glc7 in yeast; CDK inhibitor, cyclin-dependent kinase inhibitor Far1p in yeast; L3MBTL1, polycomb protein that contains malignant brain tumor (MBT) domain; CDT1, chromatin licensing and DNA replication factor 1; SET8, histone methyltransferase; XMAP215, processive microtubule polymerase; TPX2, microtubule associated protein; PLX1, polo-like kinase; DNA pol δ and η DNA polymerases delta and eta.

As one of the most abundant cellular proteins, p97 is essential for cell development, proliferation, and growth. p97 has recently emerged as a central element in the ubiquitin system involved in both proteasomal and lysosomal degradation pathways as well as ubiquitin-dependent, degradation-independent processes. A central function of p97 enzymatic activity is to convert its own ATPase activity into remodeling activity to release (extract) and process a myriad of ubiquitinated substrates from various cellular locations. p97 plays an essential role in protein homeostasis and protection from protein stress due to accumulation of short-lived, misfolded, old, and damaged proteins. The diversity of p97 related to a myriad of ubiquitinated substrates in a variety of cellular processes is governed by multiple adaptors. An adaptor hierarchy based on a second tier of p97 adaptors primarily from the UBX family controls three core p97 adaptor complexes, p97^–U^^fd1^^–Npl4^, p97^–p47^, and p97^–UBXD1^. Although the role of p97 in the cell cycle and DNA repair has been known for several decades, its function in genome stability is beginning to emerge (**Figure 4**). Elucidation of CAD orchestrated by p97 in DNA replication, DNA repair and discovery of DVC1 has finally established the role of p97 in genome stability. This was further demonstrated by discovery of a novel premature aging syndrome in a human due to a homozygotic mutation in the p97 adaptor DVC1. Although p97 overexpression correlates with tumor progression, inactivation of p97 or its adaptors leads to genome instability. These facts support the hypothesis that tight regulation of the p97 system is essential for genome stability and protection against cancer. Understanding how p97 protects cells from PICHROS and its function in DNA replication, repair, recombination, mitosis and the cell cycle will be essential for fully understanding the role of p97 in genome stability, aging and cancer. Many questions await further investigation, such as the identification of p97-substrates, adapters, and pathways that contribute to genome stability. p97 has a broad range of adaptors many of which possess ubiquitin-binding domains. This strongly suggests that p97 most probably binds and modulates variety of ubiquitinated substrates, besides the ones already known and described in this review, which are essential for genome stability. Hence, we have to identify other CAD-related substrates of p97, as well as the composition of p97-adaptor for specific substrates related to genome stability. Although it seems simple, the second tier of p97-adaptors and E3-ubiquitin ligases for many established pathways and substrates is still unknown, as indicated by question marks in **Figure 4**. In addition to its role in numerous cellular processes (**Figure 4**; fifth ring), p97 most probably also plays a role in other ubiquitin-controlled pathways and processes that directly regulate genome stability, such as base excision repair, mismatch repair, DNA damage checkpoints, and apoptosis. Finally, understanding the complex function of p97, its adaptors and substrates in genome stability might directly help to develop a new and more efficient anti-cancer drug(s). This speculation is based on effects of bortezomib (Velcade), a proteasome inhibitor currently used for treatment of multiple myeloma and mantle-cell lymphoma and fascinating results showing that p97 inhibitor significantly reduced NSCLC tumor growth in *in vitro* and *in vivo* models (Valle et al., 2011). Taken together, one anticipates interesting and dynamic time ahead in investigating the function of p97 in the context of genome stability and cancer therapy.

## Conflict of Interest Statement

The authors declare that the research was conducted in the absence of any commercial or financial relationships that could be construed as a potential conflict of interest.
